# Perceptions and experiences of the subjective well-being of people with glioblastoma: a longitudinal phenomenological study

**DOI:** 10.1093/nop/npac064

**Published:** 2022-08-18

**Authors:** Katie Sutton, Jaqualyn Moore, Jo Armes, Emma Briggs

**Affiliations:** Florence Nightingale Faculty of Nursing, Midwifery & Palliative Care, King’s College London, James Clerk Maxwell Building, 57 Waterloo Road, London, SE1 8WA, UK; Florence Nightingale Faculty of Nursing, Midwifery & Palliative Care, King’s College London, James Clerk Maxwell Building, 57 Waterloo Road, London, SE1 8WA, UK; University of Surrey, Kate Granger Building, Priestley Road, Surrey Research Park, Guildford, GU2 7YH, UK; Florence Nightingale Faculty of Nursing, Midwifery & Palliative Care, King’s College London, James Clerk Maxwell Building, 57 Waterloo Road, London, SE1 8WA, UK

**Keywords:** brain tumor, glioblastoma, phenomenology, well-being

## Abstract

**Background:**

Glioblastoma (GBM) is a devastating form of brain cancer, with a short life expectancy. In addition to this poor prognosis, people with GBM often experience symptoms that may have a profound impact on their subjective well-being (SWB). The aim of this study was to investigate the lived experiences and perceptions of people with GBM regarding their SWB.

**Methods:**

The study adopted a longitudinal, hermeneutical phenomenological approach. Twenty-seven interviews were conducted with 15 patients over a period of two years. Most participants were interviewed twice on a face-to-face basis (during combined chemotherapy and radiotherapy, and again during adjuvant chemotherapy). The hermeneutic circle was used to guide data analysis.

**Results:**

Data analysis identified four key themes that depicted the lived experiences and perceptions of SWB of people with GBM. “Experience of the disease” focuses on the impact of diagnosis, symptoms and side effects. “Daily life” relates to daily activities, family roles, work and social lives. “Coping” includes the importance of normality and goal-setting. “Experiences of care” focuses on the impact of the treatment schedule, experiences of care and impressions of the monitoring of QoL.

**Conclusion:**

SWB is affected by a variety of factors throughout the GBM disease and treatment journey. The findings of this study suggest that healthcare professionals can enhance the SWB of people with GBM by providing personalized care that supports people to set themselves goals for the future and retain a degree of normality wherever possible.

Glioblastoma (GBM) is a particularly harmful form of brain cancer, with a two-year survival rate of 18%.^1^ In addition to this poor prognosis, research has revealed that people with GBM often experience significant symptom burden resulting from both physical and cognitive deficits, which may have a profound impact on their daily life and subjective well-being (SWB).^2^ Even though subjective well-being is widely regarded as a fundamental measure of existence, the term is vague and ambiguous. It is often used interchangeably with concepts such as “health” and “quality of life,” despite the fact that it is not necessarily affected by health issues.^3^ QoL consists of numerous domains, including physical and psychological health, social relationships, and environmental aspects,^4^ whereas SWB is concerned with how people experience and evaluate their lives; involving factors such as a sense of purpose and fulfillment, and relationships with family and friends.^4^

During the last decade, there has been a significant increase in the volume of research undertaken to explore the well-being and quality of life of people diagnosed with brain tumors.^5–7^ However, on consideration of the available literature, there were some apparent “gaps” in the evidence. The aim of this study was to investigate the lived experiences and perceptions of people with GBM regarding their SWB at different points in time during the initial phases of their illness. Specifically, this study addressed omissions in the knowledge base as follows:

1. It focused specifically on people with a diagnosis of HGG rather than brain tumors in general.
2. It offered a longitudinal perspective by including data collected at different stages in the disease and treatment journey.
3. It explored the experiences and perceptions of the impact of a diagnosis of HGG on SWB using a phenomenological methodology.

These characteristics are supported by Sterckx et al,^8^ who recommended that future research should specify both type of brain tumor and the timepoint at which participants are interviewed. It is hoped that incorporating these details has helped to ensure that this study results in a unique and valuable contribution to the body of available literature.

## Methods

### Study Design

We adopted a hermeneutic phenomenological approach. This form of qualitative inquiry goes beyond description and enables interpretation of lived experience and exploration into why a person felt and responded as they did.^9^

### Population, Setting, and Sample

The population included any adult with a diagnosis of GBM receiving treatment at a cancer center in London. The Brain Tumour Unit receives tertiary referrals from both General Practitioners and hospitals across southern England, and offers a range of investigations, treatments and support for patients with brain and spinal tumors. As a result, the center has a diverse patient profile.

We excluded those <18 years, and those whose disease had progressed to a stage whereby, according to their oncologist, they did not have the mental capacity to give informed consent. Those who could not speak English fluently were also excluded, as it was felt that the essence of meaning, integral to this type of research, may have been lost if a translator was involved. These exclusion criteria were a means of limiting ethical risks related to informed consent and causing unnecessary distress to participants.

### Sample

Fifteen participants were interviewed on a maximum of three occasions. The sample was sufficient to gain new, in-depth insights into the experience of people with GBM and to allow for attrition, whilst at the same time being small enough to ensure thorough analysis in accordance with a hermeneutic phenomenological approach.^10^ A nonprobability, purposive sampling technique was used to ensure sufficient variety in age, ethnicity, and gender.

### Sample Recruitment

The researcher attended the neuro-oncology clinic weekly to identify appropriate patients. The principal researcher (KS) did not approach potential participants at the identification stage, in case this placed undue pressure on them to participate. The clinical team provided written information about the study at the end of their face-to-face appointment and sought permission for the researcher to contact potential participants. They were then given a minimum of 24 hours to consider involvement in the research. In practice, this tended to involve patients and carers taking the information sheets home to read with the agreement that the researcher would phone them before their next clinic appointment to provide further verbal information, answer any questions and to confirm their willingness to participate. This phone call also enabled the initiation of a relationship between the principal researcher and the participant. On their next attendance at the clinic written consent was taken from both the patients and participating carers by KS. Verbal consent was reiterated prior to each subsequent interview. Participants were informed that KS was an experienced oncology nurse who was undertaking the research as part of her PhD studies. KS had experience in conducting qualitative interviews with cancer patients, and had previously undertaken advanced communication skills training.

### Interviews

**Fig. 1:**
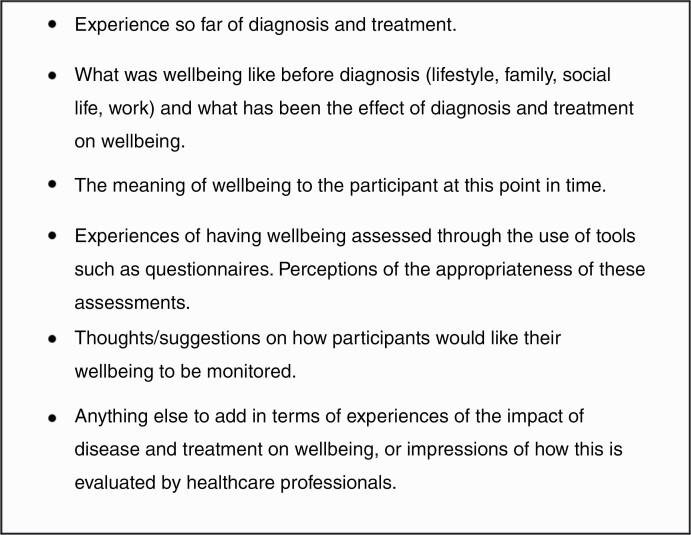
Interview guide.

Participant interviews are commonly used to collect data in phenomenological research, and generate rich descriptions of the phenomenon. Whilst phenomenology is fundamentally at odds with adopting a structured approach to interview questions, some element of interview structure is required to ensure the transferability of the research.^11^ Hence, an interview guide was developed based on prior knowledge of the field and relevant literature on SWB (Figure 1).

A core skill of the researcher is being able to adapt the language and style of interview depending on the participant. When participants started to discuss an experience or train of thought which appeared to offer a valuable insight, they were encouraged to explore this further by asking questions such as “could you tell me a bit more about that?,” “how did that make you feel?” and “what do you mean by that?”. On reflection, the use of an interview guide (to ensure all relevant topics were covered) combined with the flexibility (to permit more personalized interaction) enabled the researcher to successfully capture participants' perceptions and experiences of GBM.

All interviews were conducted in a private clinical room within the Cancer Centre at a time convenient for the participant and were audio-recorded. Interviews did not exceed one hour. All interviews were conducted by KS who was independent of the clinical teams.

Participants were invited to involve a family member and/or carer in the interview if they wished. The rationale for this decision was based on the clinical team’s view that it would offer reassurance and reduce anxiety for those who had difficulty communicating due to cognitive changes resulting from their disease.

### Longitudinal Data Collection

Collecting longitudinal data offers several significant advantages over cross-sectional studies. The primary purposes for collecting data longitudinally are to capture the depth and breadth of participants’ life experiences through the researcher’s continuing immersion in the field and to compare changes in perceptions and behavior over time.^12^ To cover the most significant changes in the disease and treatment journey for this study, the interviews were planned as follows:

1. During combined chemotherapy and radiotherapy.
2. During adjuvant chemotherapy.
3. During or shortly after any subsequent treatments (e.g., chemotherapy), or periods of stable disease.

### Ethical Considerations and Approval

Participants were undergoing treatment for a serious and life-threatening illness, therefore sensitivity, respect and the ethical principles of non-maleficence, beneficence, autonomy, and justice^13^ were the highest priority. Upsetting issues were raised on three occasions by participants. In these instances, initial advice was given by KS (an experienced Oncology Nurse) when within her scope of practice, and the clinical team was informed, with consent from the patient, to arrange a follow-up.

Confidentiality of personal data was maintained at all times in accordance with the Data Protection Act.^14^ Audio recordings of interviews were sent securely to a specialist company for transcribing. To preserve confidentiality, pseudonyms were allocated to participants and any information that may have risked breaching anonymity was removed from the transcripts. The study was reviewed and given a favorable opinion by the Brighton and Sussex NRES Committee (study code: 14/LO/1898).

### Data Analysis

Data were managed using NVivo software. To adhere to the hermeneutic circle approach to data analysis, each interview transcription was read repeatedly, with any significant statements being highlighted and allocated to an NVivo node.^15^ As themes were revisited through the re-reading and reflective stages of the hermeneutic circle process, any overlap between themes was merged, where possible, and those that no longer seemed to be justified by the data were reviewed and removed. At this stage, coding was discussed by all authors to ensure consensus on the themes.

After the initial coding of each interview, longitudinal analysis commenced. Longitudinal data analysis (LDA) was a means of exploring variation or consistencies in individual participants’ experiences at different time points in their disease and treatment.^16^

Analysis occurred within each case as well as through cross-case comparison and focused on the impact of time and changes in context on the data.^12^ Saldaña’s^12^ frequently cited guidance on analyzing longitudinal qualitative data were used as a means of ensuring a thorough and reliable analysis.

## Results

Fifteen participants (eight men and seven women) were recruited for the study and were interviewed at timepoint one. One person was approached by the clinical team, but declined participation due to the emotional burden of their recent diagnosis. Eleven of these participants were also interviewed at timepoint two. Only one participant was interviewed at timepoint three. Attrition occurred as a result of both disease progression and time restraints on the data collection period. Key characteristics of the participants are summarized in Table 1.

**Table 1:** Key Characteristics of Participants

Pseudonym	Diagnosis and Grade	Age	Gender	Ethnicity	Presence of family member/carer	Attended interview 1	Attended interview 2	Attended interview 3
*Barbara*	Glioblastoma Grade 4	45	Female	White British	Yes-daughter		Did not attend-No longer attending for treatment	
*Barry*	Glioblastoma Grade 4	47	Male	White British	No			
*Bill*	Glioblastoma Grade 4	60	Male	White British	No			
*Brian*	Glioblastoma Grade 4	64	Male	White British	Yes-wife (Ann)			
*Christopher*	Glioblastoma Grade 4	60	Male	White British	No			
*Joan*	Glioblastoma Grade 4	54	Female	White British	No			
*Kath*	Glioblastoma Grade 4	59	Female	German	No			
*Len*	Glioblastoma Grade 4	53	Male	White British	Yes-wife			
*Liu*	Glioblastoma Grade 4	68	Female	Chinese	Yes-husband		Did not attend due to disease progression	
*Maria*	Glioblastoma Grade 4	29	Female	Romanian	No			
*Mateo*	Glioblastoma Grade 4	57	Male	Spanish	No		Did not attend due to disease progression	
*Michael*	Glioblastoma Grade 4	68	Male	White British	Yes-wife and daughter			
*Reena*	Glioblastoma Grade 4	37	Female	Bengali	Interview 1 alone, interview 2 with mother			
*Tom*	Glioblastoma Grade 4	50	Male	White British	No		Did not attend due to disease progression	
*Yulia*	Glioblastoma Grade 4	41	Female	Russian	No			

**Fig. 2:**
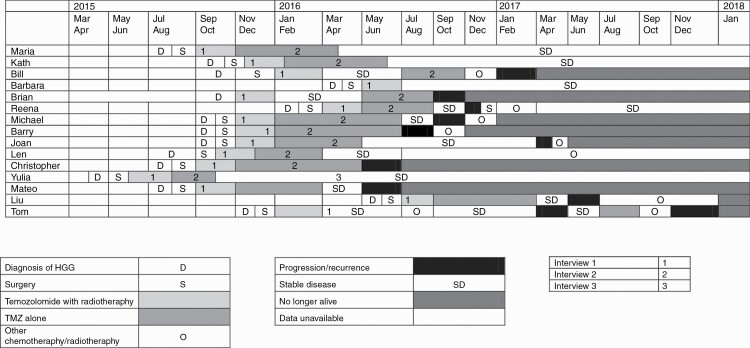
Disease and treatment duration.

By the end of the data collection period, five participants had stable disease, one was receiving second-line chemotherapy, and nine had died. A summary of disease and treatment duration for participants can be found in Figure 2.

**Fig. 3:**
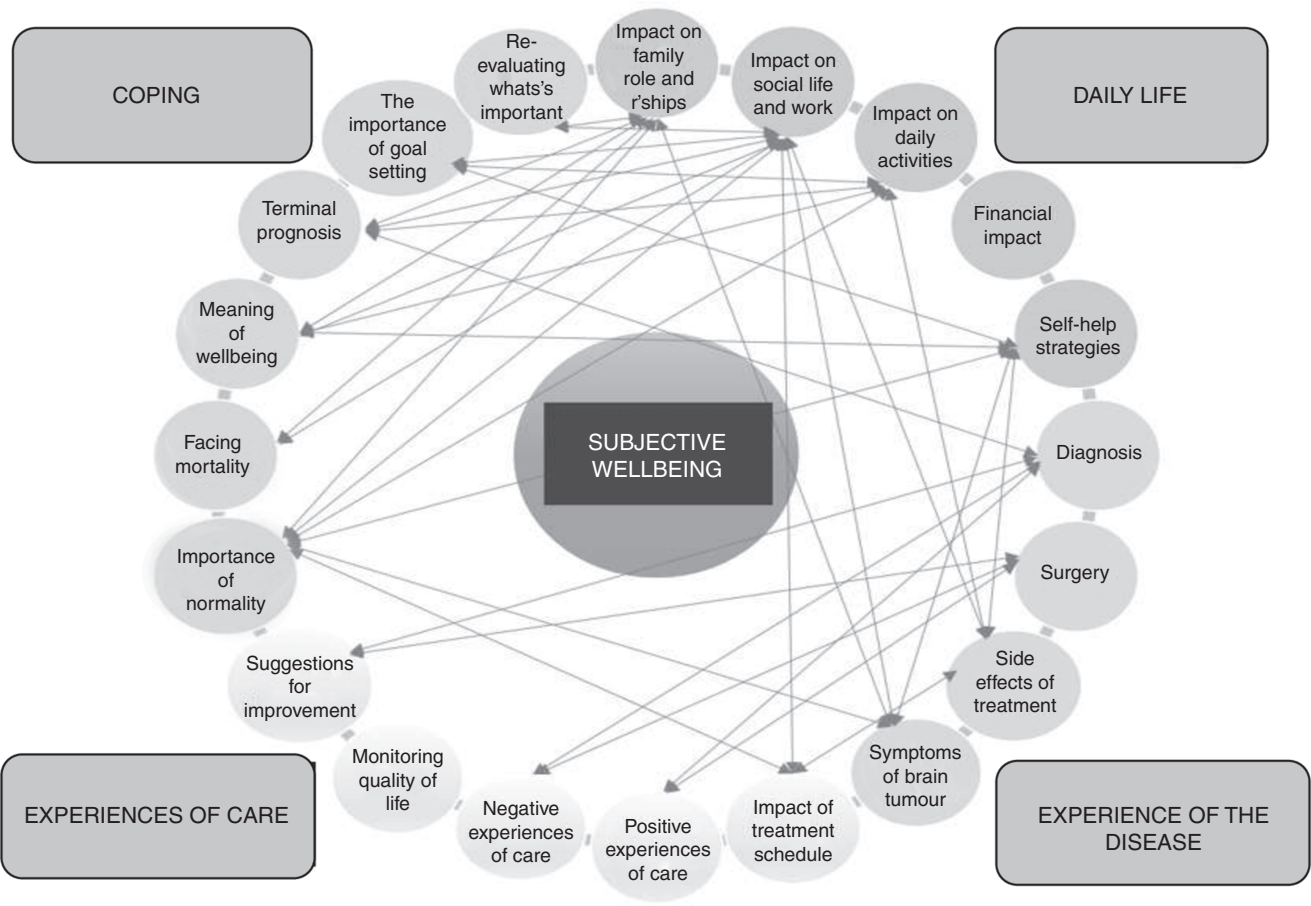
Themes and sub-themes.

The aim of this research was to explore the perceptions of people with GBM relating to the impact of their disease on their SWB over time. Data analysis identified four key themes in relation to this question. These were “experience of the disease,” “daily life,” “coping” and “experiences of care.” “Experience of the disease” focuses on the perceived impact of diagnosis, symptoms of disease and side effects of treatment on participants’ SWB. “Daily life” relates to the impact of living with GBM on participants’ daily activities, their roles within their families, and their work and social lives. The “coping” theme includes the importance of normality and the importance of goal-setting. Finally, “experiences of care” focuses on the impact of the treatment schedule and care provision on SWB. Figure 3 portrays the identified themes and sub-themes of SWB. The arrows represent associations between subthemes. Figure 3 highlights that sub-themes did not occur in isolation from one another. There were numerous interrelationships in terms of the relevance of data to multiple themes. Overlap was particularly noted between the “coping” and “experience of the disease” themes. Both the “family roles and relationships” and the “impact on social life and work” sub-themes were linked to all sub-themes in the “coping” domain, thus emphasizing the fundamental importance of family, work and social life to SWB, and their close relationship to concepts such as the importance of normality and goal-setting.

### Theme 1: Experience of the Disease

Participants spoke extensively about the shocking and almost unreal experience of being diagnosed with GBM. Emotive language and metaphors were used by participants to convey their experiences of being informed about their brain tumor:

When I was first diagnosed, it was what we are calling Black Friday, it was extremely difficult when someone shows you a scan with a great big hole in your head that is showing you that there is a big tumour and you are going to have to have something done about it pretty soon. I’d say it is absolutely life changing. Just everything like flashes before you... you think about everything and you think about nothing at the same time. It sort of consumes you completely is what I found. (Christopher)

It just – bang- you know? It was like a ton of bricks (Len’s wife Sam)

It’s like jumping on a rollercoaster …(Barry)

Diagnosis was most frequently discussed at interview one and could be seen as an “epiphany.” This was a significant event, which was at the forefront of participants' minds at this point, when the impact of the diagnosis on their SWB was at its most intense. The relevance of this then decreased as time passed and other issues became more pressing. However, there is also a possibility that the increased prevalence of the diagnosis sub-theme may have been related to the focus on this experience in interview one, which proved to be an effective way of encouraging dialogue in the early stages of the interviewer-participant relationship.

The impact on SWB of symptoms resulting from GBM was a highly prevalent theme throughout the data. For those who experienced them, seizures were a “strange” and “scary” event.

I don’t remember a thing about them, I just remember sliding into a deep hole, and that’s it. It’s a horrible feeling. (Brian)

Others reported cognitive changes that were wide-ranging at both interviews. These included issues with hearing and balance, difficulty doing up buttons and shoelaces, as well as confusion over dates and times; all of which had the potential to detrimentally affect SWB.

I think things like planning things, planning journeys…he used to plan journeys, check the timetable, do all that, and he can’t do that anymore. (Brian’s wife Ann)

The side effect mentioned most frequently by participants at both interviews was fatigue.

A lot of the time I start with something and then because I’m so tired, you know, I have to sort of give up. (Reena)

Having said this, Bill and Brian both mentioned that although they felt tired, they did not find tiredness a difficult side effect to manage.

The tiredness, it’s not like extreme weariness... I thought, I’ve heard before from cancer patients that you know oh God you are exhausted all the time. You know it is really not like that. It’s just feeling lazy. (Bill)

Although it was clear that the SWB of nine participants was detrimentally affected by treatment side effects, it cannot be ignored that six reported that they did not have any significant side effects, and were generally quite surprised by how well they were feeling.

Touch wood I’ve had no side effects as yet. (Barry)

### Theme 2: Daily Life

Two participants (Reena and Michael) discussed their feelings around a change of role in their home lives.

I don’t do anything in the house. I used to do everything. Clean the house, do the washing, looking after the whole house was my business. I want to go back to actually, you know, working in the house and not everybody fussing over me. (Reena)

Both Reena and Michael felt dissatisfied that their ability to offer a valuable contribution to family life had diminished, highlighting the importance of having a sense of purpose and “feeling useful” as a core component of SWB. Being “fussed over” or given priority within the family group, whilst understandable, was a reminder of their illness and consolidated feelings of futility.

There was a general sense amongst the participants who were mothers that they continued to put their children’s well-being ahead of their own, despite their disease, and that their role as a parent remained their top priority regardless of their own health. For example, Maria traveled back to Romania between each chemotherapy treatment to see her infant son (who was staying with her parents) regardless of how well she felt. It was vitally important to her SWB that she continued to perform her role as a mother to him, irrespective of the effort and inconvenience that this involved.

It was mentioned by seven participants at interview one that they did not feel that they wanted to return to work. There was a sense that participants identified other aspects of their lives that were more important to their SWB, such as spending time with family and pursuing hobbies.

Now I’m not doing anything, I quite enjoy it... (Brian)

I’m thinking more to stay with my family and not work too much because before I used to work long hours. But now I just want to stay with my family and enjoy more of life. Because I realise it’s very short. (Maria)

Not all of the participants saw work as having a negative effect on their SWB. By interview two, both Bill and Christopher revealed their intentions to return to some degree of working. For them work had offered a sense of purpose and satisfaction, and was therefore something that they looked forward to getting back to.

I think I might going forward want to start to do some work again, but the kind of work I want to do and when I want to do it…(Christopher)

Bill had also realized that he had developed more interest in returning to work by the time of interview two. This appeared to be linked to his desire to increase his social interactions as a means of regaining his sense of identity and a degree of normality:

I started to think well actually maybe I should go into the office for an hour or two and just start getting into it again, and I think I’d like that, like the conversations and engagement… (Bill, I2)

When asked about what was important to their SWB on a daily basis, there were striking similarities in how people chose to spend their time. Daily activities largely revolved around spending time with family, exercising, resting, being outdoors, listening to music and reading. The idea of distraction and escapism was prevalent in discussions on daily activities and SWB. This involved activities such as reading, listening to music and watching films:

First thing in the morning I generally read the paper, I do a lot of reading… It’s partly escapism but it’s partly just engaging with something. (Bill)

Barbara felt strongly that retaining a sense of control over her daily life was essential to preserving her well-being. The following extract summarizes what appeared to be an important self-help strategy for participants—to retain a sense of control in life wherever possible.

I am just getting on. I am doing the ironing, hoovering, washing, everything. It’s very important to me. Because it is the only thing I can control. I am happy and I am in control of something. I am not in control of the cancer but I am in control of something else….(Barbara)

By interview two, many participants felt an increase in their confidence and ability to improve their SWB. As well as setting themselves goals to travel and exercise more, they experienced a boost to important aspects of their SWB such as their social lives, energy levels and self-image. These were very positive changes, which heightened their sense of identity, hope and control over their lives, and were generally linked to the fact they no longer needed to attend for daily radiotherapy. As Christopher summarized:

It’s easier than the other phase, so I feel …much happier… I feel like … a bit more normal. What’s the word normal mean? But I feel I can try and do other things. (Christopher)

### Theme 3: Coping

Craving a return to normality was expressed by nine participants as something they felt significantly impacted SWB.

Most people say “I don’t want to be normal, I want to be extraordinary”…But now all I want is to do things in a normal way. (Christopher)

Analysis of this theme suggested that there was evidence of a return to what felt “more normal” by interview two:

I’m sort of getting back to normal now... I’m almost back to doing normal things, that’s what I think anyway. (Brian)

Five participants discussed the importance to their SWB of setting themselves goals for the future, and how their approach to this had altered since their diagnosis. For them, continuing to look to the future was integral to their ability to cope. For example, Barbara revealed that focusing on her children’s futures helped her to remain positive. Conversely, Yulia and Bill both felt that limiting aspirations to the short term was the best way to preserve their SWB. It seemed that for them looking too far ahead was a daunting and scary prospect.

As a coping strategy I have taken the view that we are not going to think too far ahead. (Bill)

### Theme 4: Experiences of care

It became clear during the interviews that the intensity of the treatment schedule during the combined chemotherapy and radiotherapy treatment phase had a significant impact on the SWB and daily life of the participants. Participants discussed the burden of this schedule and likened it to a “workload” and a “full time job.”

*I’m actually so busy with the treatment … you can hardly do anything …*(Yulia)

I find that it’s almost like going to work … it’s having to travel in everyday and it has become my new routine…(Kath)

Comments regarding the scheduling of treatment implied a significant improvement in SWB at interview two (once radiotherapy was completed) as participants had more time to spend on activities which enhanced their SWB such as exercise and spending time with family. Joan, Kath and Christopher commented on the relief of finishing radiotherapy, and the liberation they felt only needing to go to hospital once per treatment cycle and taking chemotherapy tablets for only 5 days every 4 weeks.

*Another great thing about it is you only do 5 days and then the next 23 you don’t do anything. That’s marvellous… that’s fantastic. I don’t have to do anything but take my pill that’s it.* (Joan)

There was considerable praise for the hospital where participants were receiving their treatment. For example, Michael expressed his gratitude for being treated as an individual:

*The hospital I can’t really fault them you know; I think they do everything they can and they do make you feel as if you’re a person rather than a number…*(Michael)

There was a general impression that participants trusted those caring for them at the cancer center, and that they placed a great deal of value on this trust and its positive impact on their SWB since diagnosis.

I feel it all feels in control, I feel I’m confident with those treating me. (Christopher)

Two participants specifically mentioned having their phone calls returned, suggesting how important this specific display of trustworthy behavior is to participants and their families, and helped them to feel a degree of control over what was happening to them.

It sounds so ridiculous but such a relief to have it, is when I phoned the phone number the lady who takes the call, she does what she says she’s going to do, always without fail, so she says she’s going to phone you back, she phones you back. (Bill)

There was a significant acknowledgment of and gratitude for the Clinical Nurse Specialists (CNSs), with five participants and their families expressing how crucial they had found their support to be in managing the impact of their cancer and treatment on their SWB. Having access to a knowledgeable professional who knew them personally not only helped them to feel they were being treated as an individual but also promoted a sense of control over their disease and treatment.

The specialist nurses on that unit, again I speak most highly of…they have done things which you know I would almost have thought ‘do I have a right to ask for’ and they’ve done them. That has been great, it’s been great also for the family to be able to talk to somebody…(Bill)

There were mixed reports on the use and value of the assessment tools used by healthcare professionals to monitor well-being. In this study, these generally took the form of Holistic Needs Assessment (HNA). In the UK, HNA is recommended for use in clinical practice as a means of assessing the physical, practical, emotional, spiritual, and social needs of people with cancer.^17^ Some participants found the HNA to be a useful prompt for discussions with their CNS, whereas others thought some questions seemed irrelevant and felt they would rather have a conversation with someone who knew them.

It had things like do you have anxiety? You know I don’t know who has a brain tumour and isn’t anxious. Some of them are a bit obvious. It’s like, some are like, you know is it affecting your sex life or something? (Joan)

*There were certain factors within that questionnaire I thought why are you asking me this? It goes into like faith..religion…there was one question about, I seem to think are you currently sexually active or something and I’m thinking … well why are you asking me that in a questionnaire?* (Barry)

It’s much more useful to talk to someone than to fill the questionnaires. (Yulia)

Indeed, talking with a familiar healthcare professional on a regular basis was cited as one of the most positive aspects of their care for participants.

## Discussion

Perhaps unsurprisingly given the severity of the condition, this study found that people with GBM experienced a significant impact on their SWB. Symptoms of the disease were wide-ranging, and had the potential to detrimentally affect SWB as the sense of identity, self-confidence and independence of participants was threatened.

This study revealed the significant impact that GBM had on an individual’s role and sense of identity within the family unit. These findings echoed those of Halkett et al.,^2^ who also described participants relinquishing roles such as preparing meals or managing family finances. Some felt distressed at this increased dependence on partners, and the subsequent perception that their relationship had become one of carer and patient rather than that of equal partners.^2^ In our study, participants highlighted how their sense of identity was threatened when they felt they could no longer make basic contributions to family life. Being “fussed over,” was acknowledged for its good intentions, but ultimately served to reinforce a sense of futility and loss of role.

Our study revealed particularly interesting insights from participants who were mothers regarding the importance to their SWB of maintaining their maternal role, which echoed findings from the literature regarding sense of identity.^18^ For some women, their role as a mother remained intrinsic to their sense of identity. They were committed to safeguarding the well-being of their children above all else. Our study found that those who were mothers felt a significant benefit to their SWB when they could continue to perform their maternal role, even when they felt unwell, and they expressed a determination to maintain as much normality as possible for their children.

This study also found that participants gained considerable satisfaction from being able to do “the little things” in life such as domestic tasks, which significantly helped them to maintain a sense of purpose and identity in a similar way to the participants in a study conducted by Sterckx et al.^19^

The terms “normal” and “normality” were used on numerous occasions by participants in this study, and it was clear that there was an overwhelming desire to maintain a normal life for as long as possible as a means of preserving their SWB, a finding which is echoed elsewhere in the literature.^5,20,21^ When individuals felt uncertain about their ability to continue with activities they considered central to their normal lives, such as working and socializing, this led to feelings of despondency and a loss of identity.^2^

This study found that goal-setting was a highly individual, and often very important aspect of preserving SWB. Some participants focused on future milestones, which they found to offer hope for the future and a distraction from their disease and treatment. Others felt safer limiting their aspirations to the short-term. This finding is supported by other literature on the value of short-term goal-setting and its relevance to the preservation of hope and SWB.^22,23^

Participants in this study highlighted the importance of consistent and individualized support to their SWB, which enabled them to take back a degree of control over their disease and treatment. The presence of a consistent and trustworthy health professional was identified as being extremely important to SWB and has also been recommended by others as a means of accessing support as and when it is individually appropriate, and to help reduce feelings of uncertainty and helplessness.^2,6,21^ In our study, it was the CNSs who fulfilled this fundamental role.

The suggestion arising from this research is that people with GBM preferred informal monitoring of their well-being with someone who they felt they could trust, and who knew them well. This finding has implications for the increasing trend of digital and remote monitoring systems in cancer services. Such systems offer a number of benefits such as preventing unnecessary hospital visits and releasing clinic capacity. Despite these benefits, it is important to be aware of the value of a personal relationship with a familiar healthcare professional to the care of people with cancer. However, as the number of people with cancer diagnoses increases, this may not always be possible. There is potential for future research into the nature of monitoring the SWB of people with cancer. The potential stratification of those with the highest support needs (such as those with terminal prognoses like GBM) could offer a means of ensuring that monitoring systems are appropriately allocated.

As previously mentioned, the term “well-being” is subject to a number of interpretations and is often used interchangeably in the literature with terms such as “quality of life.” The practical implications of this disparity of understanding became evident early in the data collection phase of this study when using the term “well-being” during interviews. When conversations started with open-ended questions such as “*Tell me about how you think your diagnosis has affected your well-being*?” many participants asked for further clarification on what the term well-being meant. We quickly realized that using terms such as “day-to-day life” or “quality of life” rather than “well-being” or “subjective well-being” appeared to have more meaning for interviewees. This was also the experience of Fox and Lantz,^24^ who similarly used “day-to-day life” with participants in their seminal qualitative study exploring the QoL of people with brain tumors.

The longitudinal nature of this study meant that KS, inevitably, developed a certain degree of personal connection with participants. In many ways, this was a positive outcome, as it allowed for the development of a rapport with participants, which is seen as being beneficial to qualitative research.^2,5^ However, as Saldana^12^ highlights, the longer a researcher spends on a study, the more likely they are to feel personally involved in each case. Due to the poor prognosis of HGG, nine of the fifteen participants had died by the end of the data collection period. This was distressing to hear, particularly when reflecting on conversations discussing participants’ hopes for the future, which some did not manage to realize. The recording of reflective notes and regular discussions amongst the research team proved to be an effective strategy for managing this emotional burden, but it cannot be denied that there is a risk of distress for researchers working in emotionally-sensitive areas, and that this should be anticipated and prepared for within research teams.^2,5^

### Limitations of the Study

A limitation of this study was that second and third interviews could not be conducted with all participants as a result of both attrition due to disease progression, and time restraints on the data collection period. Whilst four participants did not attend a second interview, only one (Yulia) had a third interview. Three further patients were eligible for a third interview, but these could not be conducted due to their absence from the clinic (Len moved to a different care provider, Maria returned to her home country and Kath requested a break in attendance). Longitudinal findings may have been strengthened had more data been collected beyond the initial stages of disease and treatment. However, it is important to highlight that data collection at two time points for most participants did allow for the demonstration of changes in SWB over time, which is rarely achieved in this population.

Five study participants had family members present during their interviews. The presence of carers during interviews was allowed in this study following discussion with the medical team, as they felt it would offer reassurance and reduce anxiety for those who may have had difficulty communicating as a result of cognitive changes brought on by their disease. Whilst this decision was made in the best interests of the participants, it may have impacted participant responses. For example, they may have been conscious of protecting their family members from information or feelings that might have caused distress. Similarly, they may have felt the need to portray positivity and optimism as a means of reassuring family members.

Both Len’s wife (Sam) and Brian’s wife (Ann) were actively involved in the discussions, and often spoke on behalf of their husbands. This appeared to be a natural and comfortable situation for them, and may have been particularly helpful as both Len and Brian were experiencing cognitive impairment. However, it cannot be assumed that Len and Brian would have responded in exactly the same way as their wives, and it is not possible to tell if this would have affected the study findings.

Only those deemed well enough to participate by the medical team could be included in the study for ethical reasons. However, this may have resulted in a degree of selection bias, which could have impacted upon the results. Had people with significant cognitive impairment participated, different insights may have been revealed.

## Conclusion

This is the first study to explore the perceptions and experiences of the SWB of people with GBM using a longitudinal phenomenological approach. SWB is affected in a variety of ways throughout the GBM disease and treatment journey. People with GBM have highlighted the importance to their SWB of retaining normality wherever possible. Health professionals can contribute positively to the SWB of people with GBM by providing personalized care that supports people to set themselves goals for the future, maintain their roles within the family unit, and to participate in daily activities when appropriate.
